# Supplementary material for: Heritable epigenetic variation facilitates long-term maintenance of epigenetic and genetic variation
Source: G3 (Bethesda). 2023 Dec 19;14(2):jkad287. doi: 10.1093/g3journal/jkad287 (PMC10849368; doi:10.1093/g3journal/jkad287)
Supplement: jkad287_Supplementary_Data [file jkad287_supplementary_data.docx]

**SUPPLEMENTAL MATERIAL**


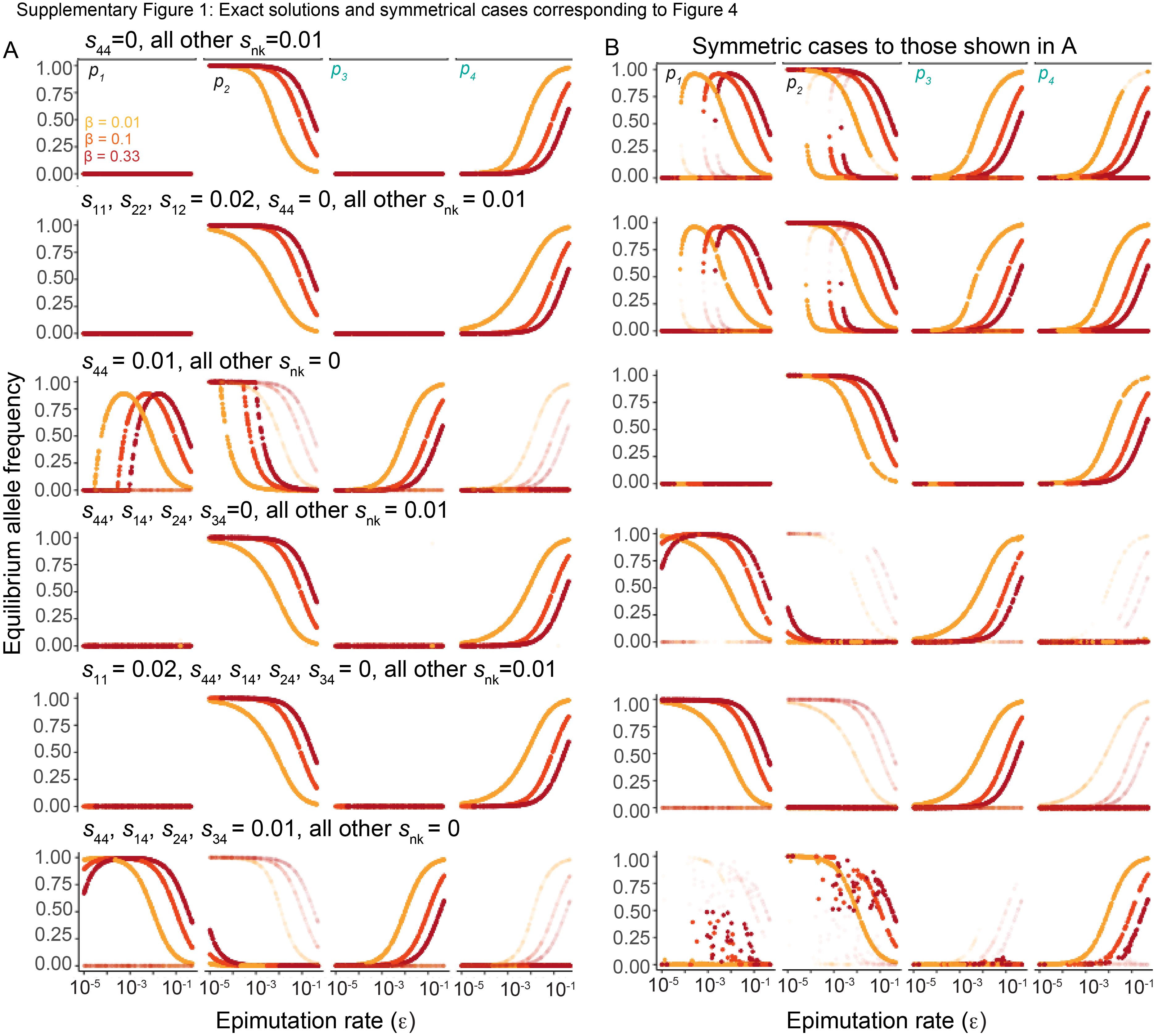


Figure S1: Exact solutions and symmetric cases corresponding to Figure 5. A-B. Equilibrium allele frequencies for *p*_1_ through *p*_4_. Stable equilibria are completely opaque points, while unstable equilibria are faded. A. From top to bottom facet, raw output for the cases shown in Figure 4 and referenced in the corresponding text with corresponding selection coefficients. B. From top to bottom facet, raw output for symmetric values to those shown A. Symmetric means that selection coefficients corresponding to *A*_1_ and *A*_3_ are swapped with *a*_2_ and *a*_4_, respectively.


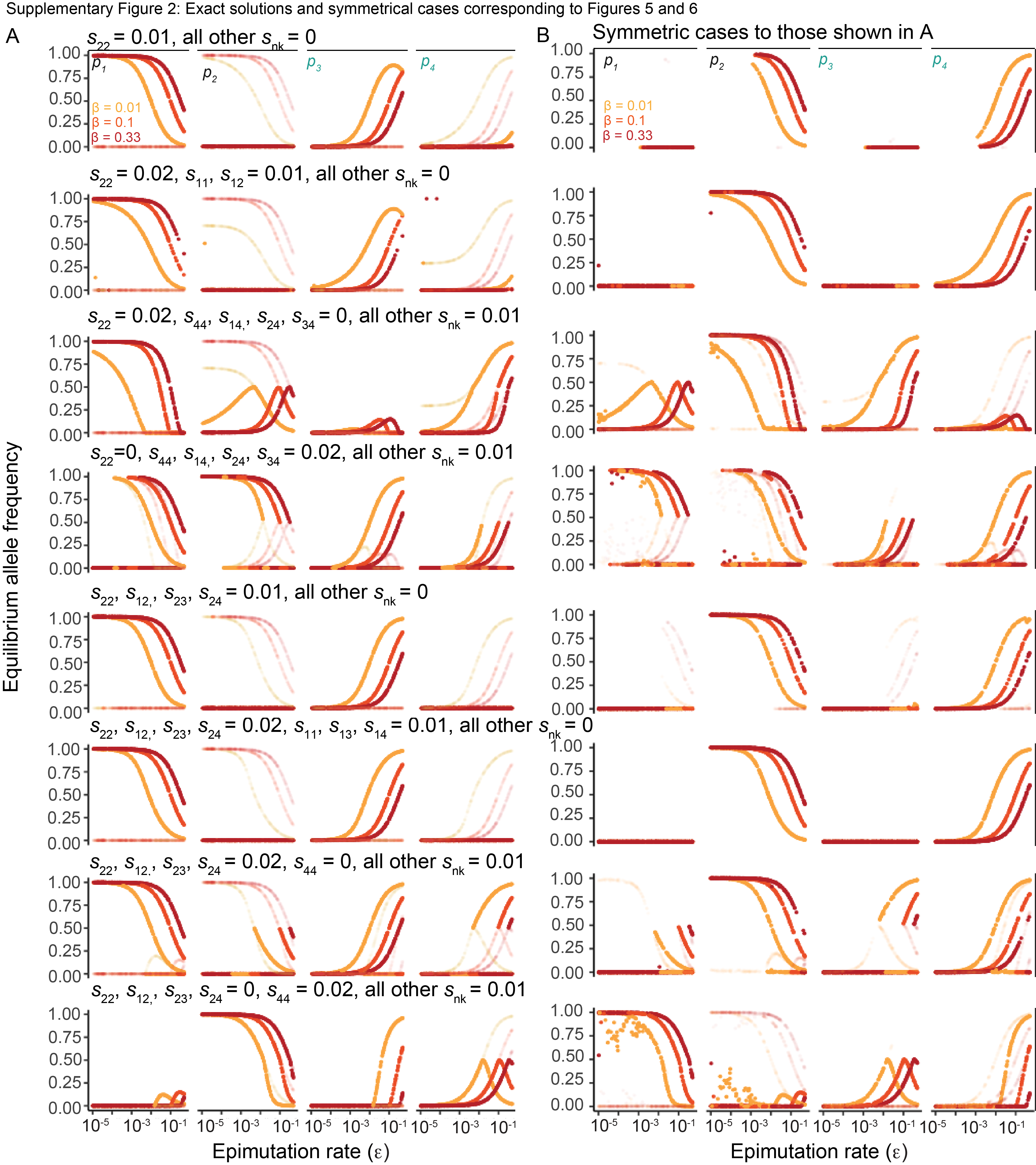


Figure S2: Exact solutions and symmetric cases corresponding to Figures 5 and 6. A-B. Equilibrium allele frequencies for *p*_1_ through *p*_4_. Stable equilibria are points with complete opacity, while unstable equilibria are faded. A. From top to bottom facet, raw output for cases shown in Figures 5 and 6 and referenced in the text, with corresponding selection coefficients. B. From top to bottom facet, raw output for symmetric values to those in A. Symmetric means that selection coefficients corresponding to *A*_1_ and *A*_3_ are swapped with *a*_2_ and *a*_4_, respectively.
